# Supplementary material for: The large mammal fossil fauna of the Cradle of Humankind, South Africa: a review
Source: PeerJ. 2025 Feb 24;13:e18946. doi: 10.7717/peerj.18946 (PMC11867040; doi:10.7717/peerj.18946)
Supplement: Supplemental Information 1 [file peerj-13-18946-s001.docx]

**Supplemental Table S1.** Sources for MNIs provided in Figure 2. Abbreviations provided in Figure 2 caption

| **Site** | **Source** |
| --- | --- |
| Cooper's Cave | (Hanon et al. 2022) |
| Drimolen | (Adams et al. 2016; Rovinsky et al. 2015) |
| Haasgat | (Adams 2012) |
| Hoogland | (Adams et al. 2010) |
| Malapa | (Val et al. 2015) |
| Sterkfontein JC | (Kibii 2004) |
| Sterkfontein MB2 | (Pickering et al. 2004) |
| Sterkfontein MB4 | (Kibii 2004) |
| Sterkfontein MB5 West | (Reynolds et al. 2007) |
| Sterkfontein Post MB6 | (Reynolds et al. 2007) |
| Sterkfontein Lincoln Cave | (Reynolds et al. 2007) |
| Swartkrans MB1 LB | (Watson 1993) |
| Swartkrans MB2 & MB3 | (Watson 1993) |
| Swartkrans MB5 | (Watson 1993) |

**References**

Adams JW. 2012. A revised listing of fossil mammals from the Haasgat cave system ex situ deposits (HGD), South Africa. *Palaeontologia Electronica* 15:1 - 88.

Adams JW, Herries AI, Hemingway J, Kegley AD, Kgasi L, Hopley P, Reade H, Potze S, and Thackeray F. 2010. Initial fossil discoveries from Hoogland, a new Pliocene primate-bearing karstic system in Gauteng Province, South Africa. *Journal of Human Evolution* 59:685-691. 10.1016/j.jhevol.2010.07.021

Adams JW, Rovinsky DS, Herries AIR, and Menter CG. 2016. Macromammalian faunas, biochronology and palaeoecology of the early Pleistocene Main Quarry hominin-bearing deposits of the Drimolen palaeocave system, South Africa. *PeerJ* 4:e1941. 10.7717/peerj.1941

Hanon R, Patou-Mathis M, Pean S, Prat S, Cohen BF, and Steininger C. 2022. Early Pleistocene hominin subsistence behaviors in South Africa: Evidence from the hominin-bearing deposit of Cooper's D (Bloubank Valley, South Africa). *Journal of Human Evolution* 162:103116. 10.1016/j.jhevol.2021.103116

Kibii JM. 2004. Comparative taxonomic, taphonomic and palaeoenvironmental analysis of 4-2.3 million year old Australopithecine cave infills at Sterkfontein.

Pickering TR, Clarke RJ, and Heaton JL. 2004. The context of Stw 573, an early hominid skull and skeleton from Sterkfontein Member 2: taphonomy and paleoenvironment. *Journal of Human Evolution* 46:279-297. 10.1016/j.jhevol.2003.12.001

Reynolds SC, Clarke JD, and Kuman K. 2007. The view from the Lincoln Cave: mid-to late Pleistocene fossil deposits from Sterkfontein hominid site, South Africa. *Journal of Human Evolution* 53:260 - 271.

Rovinsky DS, Herries AI, Menter CG, and Adams JW. 2015. First description of in situ primate and faunal remains from the Plio-Pleistocene Drimolen Makondo palaeocave infill, Gauteng, South Africa. *Palaeontologia Electronica* 18:1 - 21.

Val A, Dirks P, Backwell L, d'Errico F, and Berger LR. 2015. Taphonomic analysis of the faunal assemblage associated with the hominins (*Australopithecus sediba*) from the early Pleistocene cave deposits of Malapa, South Africa. *PLoS One* 10:e0126904. 10.1371/journal.pone.0126904

Watson V. 1993. Composition of the Swartkrans bone accumulations, in terms of skeletal parts and animals represented. In: Brain CK, ed. *Swartkrans: A cave’s chronicle of early man*. Pretoria: Transvaal Museum Monograph, 35 - 74.
