# Supplementary material for: The large mammal fossil fauna of the Cradle of Humankind, South Africa: a review
Source: PeerJ. 2025 Feb 24;13:e18946. doi: 10.7717/peerj.18946 (PMC11867040; doi:10.7717/peerj.18946)
Supplement: Supplemental Information 2 [file peerj-13-18946-s002.docx]

**Supplemental Table S2.** Taxonomic list of large mammal species at Cooper’s Cave. Integrated data from de Ruiter et al. (2009); (Hanon et al. 2019); Hanon et al. (2022)

| **Order** | **Family** | **Tribe** | **Taxon** |
| --- | --- | --- | --- |
| Primate | Hominidae |  | *Paranthropus robustus* |
|  | Cercopithecidae |  | *Cercopithecoides* sp. |
|  |  |  | *Theropithecus oswaldi* |
|  |  |  | *Papio hamadryas angusticeps* |
|  |  |  | *Papionini* sp. |
|  |  |  | *Gorgopithecus* sp. |
|  |  |  | *Gorgopithecus major* |
|  |  |  | *Parapapio* sp. |
| Carnivora | Canidae |  | *Lycaon sekowei* |
|  |  |  | *Canis mesomelas* |
|  | Felidae |  | *Dinofelis aronoki* |
|  |  |  | *Dinofelis* sp. |
|  |  |  | *Megantereon whitei* |
|  |  |  | *Panthera pardus* |
|  |  |  | *Panthera leo* |
|  |  |  | *Acynonix jubatus* |
|  |  |  | *Panthera* sp. |
|  |  |  | *Caracal caracal* |
|  |  |  | *Felis* sp. |
|  | Hyaenidae |  | *Crocuta ultra* |
|  |  |  | *Parahyaenna brunnea* |
|  |  |  | *Hyaena hyaena* |
|  |  |  | *Chasmaporthetes nitidula* |
|  |  |  | *Proteles* sp. |
| Artiodactyla | Bovidae | Alcelaphini | *Megalotragus* sp. |
|  |  |  | *Connochaetes* sp. |
|  |  |  | *Damaliscus* sp. |
|  |  | Antilopini | *Antidorcas marsupialis* |
|  |  |  | *Antidorcas recki* |
|  |  | Neotragini | *Raphicerus* sp. |
|  |  | Bovini | *Syncerus* sp. |
|  |  | Tragelaphini | *Tragelaphus strepsiceros* |
|  |  |  | *Tragelaphus scriptus* |
|  |  | Hippotragini | *Hippotragus* sp. |
|  |  | Reduncini | *Redunca fulvorufula* |
|  |  | Peleini | *Pelea* sp. |
|  | Giraffidae |  | *Sivatherium maurusium* |
|  | Suidae |  | *Metridiochoerus modestus* |
|  |  |  | *Metridiochoerus andrewsi* |
| Perissodactyla | Equidae |  | *Equus quagga* |
|  |  |  | *Equus capensis* |
|  |  |  | *Eurygnathohippus cornelianus* |

**References**

de Ruiter D, Pickering R, Steininger CM, Kramers JD, Hancox PJ, Churchill SE, Berger LR, and Backwell L. 2009. New *Australopithecus robustus* fossils and associated U-Pb dates from Cooper's Cave (Gauteng, South Africa). *Journal of Human Evolution* 56:497-513. 10.1016/j.jhevol.2009.01.009

Hanon R, Patou-Mathis M, Pean S, and Prat S. 2019. Paleobiodiversity and large mammal associations during the Late Pliocene and the Early Pleistocene in South Africa *Quaternaire* 30:243 - 256.

Hanon R, Patou-Mathis M, Pean S, Prat S, Cohen BF, and Steininger C. 2022. Early Pleistocene hominin subsistence behaviors in South Africa: Evidence from the hominin-bearing deposit of Cooper's D (Bloubank Valley, South Africa). *Journal of Human Evolution* 162:103116. 10.1016/j.jhevol.2021.103116
