# Supplementary material for: The large mammal fossil fauna of the Cradle of Humankind, South Africa: a review
Source: PeerJ. 2025 Feb 24;13:e18946. doi: 10.7717/peerj.18946 (PMC11867040; doi:10.7717/peerj.18946)
Supplement: Supplemental Information 3 [file peerj-13-18946-s003.docx]

| **Site** | **Primate publications** | **Carnivore publications** | **Bovid publications** | **General fauna publications** |
| --- | --- | --- | --- | --- |
| Bolt’s Farm | (Elton 2001; Freedman 1965; Gilbert 2007; Gommery et al. 2014; Gommery et al. 2008; Gommery et al. 2009; Williams et al. 2007) | (Broom 1939; Cooke 1991) | (Cooke 1996; Reynolds 2007; van Zyl et al. 2016) | (Badenhorst et al. 2011) |
| Cooper’s Cave | (DeSilva et al. 2013; Folinsbee & Reisz 2013; Val et al. 2014) | (Cohen et al. 2019; Hartstone-Rose et al. 2007; Hartstone-Rose et al. 2010; Kuhn et al. 2017; O'Regan et al. 2013; O'Regan & Steininger 2017) | (Steininger 2011)  (Hanon et al. 2022) |  |
| Drimolen |  | (O’Regan & Menter 2009) |  | (Adams et al. 2016; Rovinsky et al. 2015) |
| Haasgat | (McKee & Keyser 1994; McKee et al. 2011) |  |  | (Adams 2012; Keyser 1991; Plug & Keyser 1994) |
| Hoogland | (Adams et al. 2010) |  |  |  |
| Malapa | (Gilbert et al. 2015) | (Kuhn et al. 2016; Kuhn et al. 2011; van der Merwe et al. 2021) | (Brophy et al. 2016) | (Val et al. 2015) |
| Sterkfontein | (Codron et al. 2005; Elton 2001; Freedman 1976; Freedman & Stenhouse 1972; Heaton 2006; Jones 1937; McKee 1993; Mokokwe 2016; Thackeray & Myer 2004; Williams et al. 2007) | (Ewer 1955a; Ewer 1955b; Ewer 1955c; Ewer 1956a; Ewer 1956b; Ewer 1956c; O'Regan 2007; O'Regan & Reynolds 2009; Turner 1987; Turner 1997) | (Sambo 2020; van der Merwe & Thackeray 1997; Vrba 1974a; Vrba 1974b; Vrba 1974c; Vrba 1975) | (Kibii 2004; Ogola 2009; Pickering et al. 2004) |
| Swartkrans | (Codron et al. 2005; Delson 1988; Elton 2001; Freedman & Brain 1977; Lee-Thorp et al. 1989; Pickering et al. 2017) | (Ewer 1955a; Ewer 1955b; Ewer 1955c; Ewer 1956a; Ewer 1956b; Ewer 1956c; Hendey 1974; Turner 1993) | (Sambo 2020; Steininger 2011; Vrba 1974d; Vrba 1974e; Vrba 1975) | (de Ruiter 2003; Watson 1993) |

**Supplemental Table S3.** Each publication included in the creation of Figure 3

**References**

Adams JW. 2012. A revised listing of fossil mammals from the Haasgat cave system ex situ deposits (HGD), South Africa. *Palaeontologia Electronica* 15:1 - 88.

Adams JW, Herries AI, Hemingway J, Kegley AD, Kgasi L, Hopley P, Reade H, Potze S, and Thackeray F. 2010. Initial fossil discoveries from Hoogland, a new Pliocene primate-bearing karstic system in Gauteng Province, South Africa. *Journal of Human Evolution* 59:685-691. 10.1016/j.jhevol.2010.07.021

Adams JW, Rovinsky DS, Herries AIR, and Menter CG. 2016. Macromammalian faunas, biochronology and palaeoecology of the early Pleistocene Main Quarry hominin-bearing deposits of the Drimolen palaeocave system, South Africa. *PeerJ* 4:e1941. 10.7717/peerj.1941

Badenhorst S, Senegas F, Gommery D, Potze S, Kgasi L, and Thackeray F. 2011. Pleistocene faunal remains from Garage Ravine cave, Bolt's Farm in the Cradle of Humankind World Heritage Site area, Gauteng South Africa. *Annals of the Ditsong National Museum of Natural History* 1:33 - 40.

Broom R. 1939. A preliminary account of the Pleistocene carnivores of the Transvaal Caves. *Annals of the Transvaal Museum* 19:331 - 338.

Brophy JK, de Ruiter D, Fortelius M, Bamford M, and Berger LR. 2016. Pleistocene Bovidae (Mammalia) from Malapa, Gauteng province, South Africa. *Palaeontologia Electronica* 19:1 - 22.

Codron D, Luyt CJ, Lee-Thorp J, Sponheimer M, de Ruiter D, and Codron J. 2005. Utilization of savanna-based resources by Plio-Pleistocene baboons. *South African Journal of Science* 101:245 - 248.

Cohen BF, O'Regan HJ, and Steininger C. 2019. Mongoose Manor: Herpestidae remains from the Early Pleistocene Cooper’s D locality in the Cradle of Humankind, Gauteng, South Africa. *Palaeontologia Africana* 53:97 - 113.

Cooke H. 1991. *Dinofelis barlowi* (Mammalia, Carnivora, Felidae) cranial material from Bolt’s Farm, collected by the University of California African expedition. *Palaeontologia Africana* 28:9-21.

Cooke H. 1996. Sexual dimorphism in *Antidorcas recki* from Bolt’s Farm, South Africa, in the University of California collections. In: Stewart K, and Seymour K, eds. *Palaeoecology and palaeoenvironments of Late Cenozoic Mammals*. Toronto: University of Toronto Press, 537-553.

de Ruiter D. 2003. Revised faunal lists for Members 1-3 of Swartkrans, South Africa. *Annals of the Transvaal Museum* 40:29 - 41.

Delson E. 1988. Chronology of South African australopith site units. In: Grine FE, ed. *Evolutionary history of the “robust” australopithecines*. New York: Aldine de Gruyter, 317-324.

DeSilva JM, Steininger CM, and Patel BA. 2013. Cercopithecoid primate postcranial fossils from Cooper's D, South Africa. *Geobios* 46:381-394. 10.1016/j.geobios.2013.07.001

Elton S. 2001. Locomotor and habitat classifications of cercopithecoid postcranial material from Sterkfontein Member 4, Bolt's Farm and Swartkrans Members 1 and 2, South Africa. *Palaeontologia Africana* 37.

Ewer R. 1955a. The fossil carnivores of the Transvaal caves. The Hyaenidae, other than Lycyaena, of Swartkrans and Sterkfontein. *Proceedings of the Zoological Society of London* 124:815-837.

Ewer R. 1955b. The fossil carnivores of the Transvaal caves. The lycyaenas of Sterkfontein and Swartkrans, together with some general considerations of the Transvaal fossil hyaenids. *Proceedings of the Zoological Society of London* 124:839-857.

Ewer R. 1955c. The fossil carnivores of the Transvaal caves: Machairodontinae. *Proceedings of the Zoological Society of London* 125:587-615.

Ewer R. 1956a. The fossil carnivores of the Transvaal caves: Canidae. *Proceedings of the Zoological Society of London* 126:97-120.

Ewer R. 1956b. The fossil carnivores of the Transvaal Caves: FeIinae. *Proceedings of the Zoological Society of London* 126:83-95.

Ewer R. 1956c. The fossil carnivores of the Transvaal caves: two new viverrids, together with some general considerations. *Proceedings of the Zoological Society of London* 126:259-274.

Folinsbee KE, and Reisz RR. 2013. New craniodental fossils of papionin monkeys from Cooper's D, South Africa. *Am J Phys Anthropol* 151:613-629. 10.1002/ajpa.22317

Freedman L. 1965. Fossil and subfossil primates from the limestone deposits at Taung, Bolt's Farm and Witkrans, South Africa. *Palaeontologia Africana* 9:19 - 48.

Freedman L. 1976. South African Fossil Cercopithecoidea: A Re-assessment Including a Description of New Material from Makapansgat, Sterkfontein and Taung. *Journal of Human Evolution* 5:297-315.

Freedman L, and Brain CK. 1977. A re-examination of the cercopithecoid fossils from Swartkrans (Mammalia: Cercopithecidae). *Annals of the Transvaal Museum* 30:211 - 218.

Freedman L, and Stenhouse NS. 1972. The *Parapapio* species of Sterkfontein, Transvaal, South Africa. *Palaeontologia Africana*:93 - 111.

Gilbert CC. 2007. Identification and description of the first *Theropithecus* (Primates: Cercopithecidae) material from Bolt's Farm, South Africa. *Annals of the Transvaal Museum* 44:1-10.

Gilbert CC, Steininger CM, Kibii JM, and Berger LR. 2015. *Papio* cranium from the hominin-bearing site of Malapa: Implications for the evolution of modern baboon cranial morphology and South African Plio-Pleistocene biochronology. *PLoS One* 10:e0133361. 10.1371/journal.pone.0133361

Gommery D, Sénégas F, and Thackeray F. 2014. Cercopithecoidea material from the Middle Pliocene site, Waypoint 160, Bolt's Farm, South Africa. *Annals of the Ditsong National Museum of Natural History* 4:1-8.

Gommery D, Thackeray F, Senegas F, Potze S, and Kgasi L. 2008. The earliest primate (*Parapapio* sp.) from the Cradle of Humankind World Heritage site (Waypoint 160, Bolt’s Farm, South Africa). *South African Journal of Science* 104:405 - 408.

Gommery D, Thackeray J, Sénégas F, Potze S, and Kgasi L. 2009. Additional fossils of *Parapapio* sp. from Waypoint 160 (Bolt's Farm, South Africa), dated between 4 and 4.5 million years ago. *Annals of the Transvaal Museum* 46:63-72.

Hanon R, Pean S, Prat S, Rector AL, and Steininger C. 2022. Fossil Bovidae from the Hominini-bearing site of Cooper’s D (Bloubank Valley, South Africa): implications for *Paranthropus robustus* Broom, 1938 and early *Homo* Linnaeus, 1758 habitat preferences. *Comptes Rendus Palevol* 21:431 - 450.

Hartstone-Rose A, de Ruiter D, Berger LR, and Churchill SE. 2007. A sabre-tooth felid from Coopers Cave (Gauteng, South Africa) and its implications for *Megantereon* (Felidae: Machairodontinae) taxonomy. *Palaeontologia Africana* 42:99 - 108.

Hartstone-Rose A, Werdelin L, de Ruiter D, Berger LR, and Churchill SE. 2010. The Plio-Pleistocene ancestor of wild dogs, *Lycaon sekowei* N. SP. *Journal of Paleontology* 84:299 - 308.

Heaton JL. 2006. Taxonomy of the Sterkfontein fossil Cercopithecinae: the *Papionini* of members 2 and 4 (Gauteng, South Africa). Indiana University.

Hendey QB. 1974. New fossil carnivores from the Swartkrans australopithecine site (Mammalia: Carnivora). *Annals of the Transvaal Museum* 29.

Jones TR. 1937. A new fossil primate from Sterkfontein, Krugersdorp, Transvaal. *South African Journal of Science* 33:709 - 728.

Keyser AW. 1991. The palaeontology of Haasgat: A preliminary account. *Palaeontologia Africana* 28:29 - 33.

Kibii JM. 2004. Comparative taxonomic, taphonomic and palaeoenvironmental analysis of 4-2.3 million year old Australopithecine cave infills at Sterkfontein.

Kuhn BF, Hartstone-Rose A, Lacruz RS, Herries AIR, Werdelin L, Bamford MK, and Berger LR. 2016. The carnivore guild circa 1.98 million years: biodiversity and implications for the palaeoenvironment at Malapa, South Africa. *Palaeobiodiversity and Palaeoenvironments* 96:611-616. 10.1007/s12549-016-0245-0

Kuhn BF, Werdelin L, Hartstone-Rose A, Lacruz RS, and Berger LR. 2011. Carnivoran remains from the Malapa hominin site, South Africa. *PLoS One* 6:e26940. 10.1371/journal.pone.0026940

Kuhn BF, Werdelin L, and Steininger C. 2017. Fossil Hyaenidae from Cooper’s Cave, South Africa, and the palaeoenvironmental implications. *Palaeobiodiversity and Palaeoenvironments* 97:355-365. 10.1007/s12549-016-0247-y

Lee-Thorp J, Van der Merwe NJ, and Brain CK. 1989. Isotopic evidence for dietary differences between two extinct baboon species from Swartkrans. *Journal of Human Evolution* 18:183 - 189.

McKee JK. 1993. Taxonomic and evolutionary affinities of *Papio izodi* fossils from Taung and Sterkfontein. *Palaeontologia Africana* 30:43 - 49.

McKee JK, and Keyser AW. 1994. Craniodental remains of *Papio angusticeps* from the Haasgat cave site, South Africa. *International Journal of Primatology* 15:823 - 841.

McKee JK, von Mayer A, and Kuykendall KL. 2011. New species of Cercopithecoides from Haasgat, North West Province, South Africa. *Journal of Human Evolution* 60:83-93. 10.1016/j.jhevol.2010.08.002

Mokokwe DW. 2016. Taxonomy, taphonomy, and spatial distribution of the Cercopithecoid postcranial fossils from Sterkfontein caves Ph.D. University of the Witwatersrand.

O'Regan HJ. 2007. Revision of the Carnivora from Member 5, Sterkfontein, South Africa, based on a re-assessment of the published material and site stratigraphy. *Annals of the Transvaal Museum* 44:209 - 214.

O'Regan HJ, Cohen BF, and Steininger C. 2013. Mustelid and viverrid remains from the Pleistocene site of Cooper’s D, Gauteng, South Africa. *Palaeontologia Africana* 48:19–23.

O'Regan HJ, and Reynolds SC. 2009. An ecological reassessment of the southern African carnivore guild: a case study from Member 4, Sterkfontein, South Africa. *J Hum Evol* 57:212-222. 10.1016/j.jhevol.2009.04.002

O'Regan HJ, and Steininger C. 2017. Felidae from Cooper's Cave, South Africa (Mammalia: Carnivora). *Geodiversitas* 39:315-332. 10.5252/g2017n2a8

O’Regan HJ, and Menter CG. 2009. Carnivora from the Plio-Pleistocene hominin site of Drimolen, Gauteng, South Africa. *Geobios* 42:329-350. 10.1016/j.geobios.2009.03.001

Ogola CA. 2009. The Sterkfontein western Breccias: Stratigraphy, fauna and artefacts Ph.D. University of the Witwatersrand.

Pickering TR, Clarke RJ, and Heaton JL. 2004. The context of Stw 573, an early hominid skull and skeleton from Sterkfontein Member 2: taphonomy and paleoenvironment. *Journal of Human Evolution* 46:279-297. 10.1016/j.jhevol.2003.12.001

Pickering TR, Heaton JL, Throckmorton ZJ, Prang TC, and Brain C. 2017. A burned primate cuboid from Swartkrans Cave, South Africa. *Annals of the Ditsong National Museum of Natural History* 7:1-7.

Plug I, and Keyser AW. 1994. Haasgat cave, a Pleistocene site in the central Transvaal: Geomorphological, faunal and taphonomic considerations. *Annals of the Transvaal Museum* 39:139 - 145.

Reynolds SC. 2007. Temporal variation in Plio-Pleistocene *Antidorcas* (Mammalia: Bovidae) horncores: the case from Bolt’s Farm and why size matters. *South African Journal of Science* 103:47 - 50.

Rovinsky DS, Herries AI, Menter CG, and Adams JW. 2015. First description of in situ primate and faunal remains from the Plio-Pleistocene Drimolen Makondo palaeocave infill, Gauteng, South Africa. *Palaeontologia Electronica* 18:1 - 21.

Sambo R. 2020. Taxon-free ecomorphological analysis of fossil bovids from the Sterkfontein and Swartkrans deposits, South Africa.

Steininger C. 2011. The dietary behaviour of early Pleistocene bovids from Cooper’s Cave and Swartkrans, South Africa.

Thackeray J, and Myer S. 2004. *Parapapio broomi* and *Parapapio jonesi* from Sterkfontein: males and females of one species? *Annals of the Transvaal Museum* 41:79-82.

Turner A. 1987. New fossil carnivore remains from Sterkfontein hominid site (Mammalia: Carnivora). *Annals of the Transvaal Museum* 34.

Turner A. 1993. New fossil carnivore remains from Swartkrans. In: Brain CK, ed. *Swartkrans: a cave’s chronicle of early man*. Pretoria: Transvaal Museum, 151-166.

Turner A. 1997. Further remains of Carnivora (Mammalia) from the Sterkfontein hominid site. *Palaeontologia Africana* 34:115 - 126.

Val A, Dirks P, Backwell L, d'Errico F, and Berger LR. 2015. Taphonomic analysis of the faunal assemblage associated with the hominins (*Australopithecus sediba*) from the early Pleistocene cave deposits of Malapa, South Africa. *PLoS One* 10:e0126904. 10.1371/journal.pone.0126904

Val A, Taru P, and Steininger C. 2014. New taphonomic analysis of large-bodied primate assemblage from Cooper's D, Bloubank Valley, South Africa. *South African Archaeological Bulletin* 69:49 - 58.

van der Merwe NJ, and Thackeray F. 1997. Stable carbon isotope analysis of Plio-Pleistocene ungulate teeth from Sterkfontein, South Africa. *South African Journal of Science* 93.

van der Merwe RH, Baker SE, and Kuhn BF. 2021. New Viverridae specimens from the Malapa hominin site and their utility as palaeoenvironmental indicators. *Geobios* 68:109-123. 10.1016/j.geobios.2021.04.005

van Zyl W, Badenhorst S, and Brink JS. 2016. Pleistocene Bovidae from X Cave on Bolt's Farm in the Cradle of Humankind in South Africa. *Annals of the Ditsong National Museum of Natural History* 6:39 - 71.

Vrba E. 1974a. Chronological and ecological implications of the fossil Bovidae at the Sterkfontein Australopithecine site. *Nature* 250:19 - 23.

Vrba E. 1974b. Description and taxonomy of the Sterkfontein extension West pit (SE) Bovidae. *Transvaal Museum Memoirs* 21:49 - 52.

Vrba E. 1974c. Description and taxonomy of the Sterkfontein type locality (STS) Bovidae. *Transvaal Museum Memoirs* 21:43 - 48.

Vrba E. 1974d. Description and taxonomy of the Swartkrans Member 1 (SKa) Bovidae. *Transvaal Museum Memoirs* 21.

Vrba E. 1974e. Description and taxonomy of the Swartkrans Member 2 (SKa) Bovidae. *Transvaal Museum Memoirs* 21:22 - 30.

Vrba E. 1975. Some evidence of chronology and palaeoecology of Sterkfontein, Swartkrans and Kromdraai from the fossil Bovidae. *Nature* 254:301-304.

Watson V. 1993. Composition of the Swartkrans bone accumulations, in terms of skeletal parts and animals represented. In: Brain CK, ed. *Swartkrans: A cave’s chronicle of early man*. Pretoria: Transvaal Museum Monograph, 35 - 74.

Williams FL, Ackermann RR, and Leigh SR. 2007. Inferring Plio-Pleistocene southern African biochronology from facial affinities in Parapapio and other fossil papionins. *Am J Phys Anthropol* 132:163-174. 10.1002/ajpa.20504
