# Supplementary material for: The large mammal fossil fauna of the Cradle of Humankind, South Africa: a review
Source: PeerJ. 2025 Feb 24;13:e18946. doi: 10.7717/peerj.18946 (PMC11867040; doi:10.7717/peerj.18946)
Supplement: Supplemental Information 4 [file peerj-13-18946-s004.docx]

**Supplemental Table S4.** Taxonomic list of large mammal species at Drimolen. Integrated data from Adams et al. (2016); Hanon et al. (2019); Rovinsky et al. (2015)

| **Order** | **Family** | **Tribe** | **Taxon** | **DMQ** | **DMK** |
| --- | --- | --- | --- | --- | --- |
| Primate | Hominidae |  | *Paranthropus robustus* | X |  |
|  |  |  | *Homo erectus* | X |  |
|  | Cercopithecidae |  | *Cercopithecoides williamsi* | X |  |
|  |  |  | *Cercopithecoides* sp. |  | X |
|  |  |  | *Papio hamadryas robinsoni* | X |  |
| Carnivora | Canidae |  | *Vulpes chama* | X | X |
|  |  |  | *Canis* sp. | X |  |
|  | Felidae |  | *Dinofelis piveteaui* | X |  |
|  |  |  | *Dinofelis barlowi* | X |  |
|  |  |  | *Dinofelis* sp. | X | X |
|  |  |  | *Megantereon whitei* | X |  |
|  |  |  | *Panthera pardus* | X |  |
|  |  |  | *Panthera* sp. | X |  |
|  |  |  | *Felis silvestris lybica* | X |  |
|  |  |  | *Caracal caracal* | X |  |
|  | Hyaenidae |  | *Chasmaporthetes nitidula* | X | X |
|  |  |  | *Chasmaporthetes silberbergi* | X |  |
| Artiodactyla | Bovidae | Alcelaphini | *Megalotragus* sp. | X |  |
|  |  |  | *Connochaetes* sp. | X |  |
|  |  |  | *Damaliscus* sp. | X |  |
|  |  |  | Alcelaphini indet. |  | X |
|  |  | Antidorcas | *Antidorcas recki* | X |  |
|  |  |  | Antilopini indet. |  | X |
|  |  | Neotragini | *Raphicerus* sp. | X |  |
|  |  | Tragelaphini | *Tragelaphus* sp. | X |  |
|  |  | Reduncini | *Redunca fulvorufula* | X |  |
|  |  | Hippotragini | *Hippotragus* sp. |  | X |
|  |  | Cephalophini | *Oreotragus* sp. | X |  |
|  | Suidae |  | *Metridiochoerus* sp. |  | X |
| Perissodactyla | Equidae |  | *Equus quagga* | X |  |
|  |  |  | *Eurygnathohippus cornelianus* |  | X |

* DMQ = Main Quarry, DMK = Makondo

**References**

Adams JW, Rovinsky DS, Herries AIR, and Menter CG. 2016. Macromammalian faunas, biochronology and palaeoecology of the early Pleistocene Main Quarry hominin-bearing deposits of the Drimolen palaeocave system, South Africa. *PeerJ* 4:e1941. 10.7717/peerj.1941

Hanon R, Patou-Mathis M, Pean S, and Prat S. 2019. Paleobiodiversity and large mammal associations during the Late Pliocene and the Early Pleistocene in South Africa *Quaternaire* 30:243 - 256.

Rovinsky DS, Herries AI, Menter CG, and Adams JW. 2015. First description of in situ primate and faunal remains from the Plio-Pleistocene Drimolen Makondo palaeocave infill, Gauteng, South Africa. *Palaeontologia Electronica* 18:1 - 21.
