# Supplementary material for: The large mammal fossil fauna of the Cradle of Humankind, South Africa: a review
Source: PeerJ. 2025 Feb 24;13:e18946. doi: 10.7717/peerj.18946 (PMC11867040; doi:10.7717/peerj.18946)
Supplement: Supplemental Information 5 [file peerj-13-18946-s005.docx]

**Supplemental Table S5.** Taxonomic list of large mammal species at Haasgat. Integrated data from Adams (2012); Adams & Rovinsky (2018); Hanon et al. (2019)

| **Order** | **Family** | **Tribe** | **Taxon** |
| --- | --- | --- | --- |
| Primate | Cercopithecidae |  | *Cercopithecoides williamsi* |
|  |  |  | *Cercopithecoides haasgati* |
|  |  |  | *Cercopithecoides coronatus* |
|  |  |  | *Cercopithecoides* sp. |
|  |  |  | *Papio hamadryas angusticeps* |
|  |  |  | *Parapapio* sp. |
| Carnivora | Felidae |  | *Dinofelis* sp. |
| Artiodactyla | Bovidae | Alcelaphini | *Megalotragus* sp. |
|  |  |  | *Connochaetes gnou* |
|  |  |  | *Connochaetes* sp. |
|  |  |  | *Damaliscus dorcas* |
|  |  |  | *Damaliscus* sp. |
|  |  | Antilopini | *Antidorcas marsupialis* |
|  |  |  | *Antidorcas bondi* |
|  |  |  | *Antidorcas* sp. |
|  |  | Tragelaphini | *Tragelaphus* sp. |
|  |  |  | *Taurotragus* sp. |
|  |  | Hippotragini | *Hippotragus* sp. |
|  |  | Cephalophini | *Oreotragus* sp. |
|  |  | Reduncini | Reduncini indet. |
| Perissodactyla | Equidae |  | *Equus quagga* |
|  |  |  | *Equus capensis* |
|  |  |  | *Equus* sp. |

**References**

Adams JW. 2012. A revised listing of fossil mammals from the Haasgat cave system ex situ deposits (HGD), South Africa. *Palaeontologia Electronica* 15:1 - 88.

Adams JW, and Rovinsky DS. 2018. Taphonomic interpretations of the Haasgat HGD assemblage: A case study in the impact of sampling and preparation methods on reconstructing South African karstic assemblage formation. *Quaternary International* 495:4-18. 10.1016/j.quaint.2018.01.036

Hanon R, Patou-Mathis M, Pean S, and Prat S. 2019. Paleobiodiversity and large mammal associations during the Late Pliocene and the Early Pleistocene in South Africa *Quaternaire* 30:243 - 256.
