# Supplementary material for: The large mammal fossil fauna of the Cradle of Humankind, South Africa: a review
Source: PeerJ. 2025 Feb 24;13:e18946. doi: 10.7717/peerj.18946 (PMC11867040; doi:10.7717/peerj.18946)
Supplement: Supplemental Information 6 [file peerj-13-18946-s006.docx]

**Supplemental Table S6.** Taxonomic list of large mammal species at Hoogland. Integrated data from Adams et al. (2010); Broom (1936); Freedman (1957)

| **Order** | **Family** | **Tribe** | **Taxon** |
| --- | --- | --- | --- |
| Primate | Cercopithecidae |  | *Theropithecus oswaldi* |
|  |  |  | *Dinopithecus ingens* |
|  |  |  | *Papio robinsoni* |
| Artiodactyla | Bovidae | Alcelaphini | Alcelaphini indet. |
|  |  | Antilopini | *Antidorcas bondi* |
|  |  |  | *Antidorcas recki* |
|  |  |  | *Antidorcas* sp. |
|  |  | Cephalophini | Cephalophini indet. |
|  |  | Neotragini | *Oreotragus oreotragus* |
|  |  | Reduncini | *Kobus* sp. |
|  |  |  | *Redunca* sp. |
|  |  | Tragelaphini | *Tragelaphus strepsiceros* |
|  |  |  | *Tragelaphus* sp. |

* This list assumes that Schurveberg and Hoogland are the same site

**References**

Adams JW, Herries AI, Hemingway J, Kegley AD, Kgasi L, Hopley P, Reade H, Potze S, and Thackeray F. 2010. Initial fossil discoveries from Hoogland, a new Pliocene primate-bearing karstic system in Gauteng Province, South Africa. *Journal of Human Evolution* 59:685-691. 10.1016/j.jhevol.2010.07.021

Broom R. 1936. Letter to Mr. Hausleitner, Schruveberg (Hennops River), Pretoria. 05/19/1936. Transvaal Museum Document #TM 17/35.

Freedman L. 1957. The fossil cercopithecoidea of South Africa. *Annals of the Transvaal Museum* 23:121-262.
