# Supplementary material for: The large mammal fossil fauna of the Cradle of Humankind, South Africa: a review
Source: PeerJ. 2025 Feb 24;13:e18946. doi: 10.7717/peerj.18946 (PMC11867040; doi:10.7717/peerj.18946)
Supplement: Supplemental Information 7 [file peerj-13-18946-s007.docx]

**Supplemental Table S7.** Taxonomic list of large mammal species at Malapa. Integrated data from Brophy et al. (2016); Dirks et al. (2010); Hanon et al. (2019); Val et al. (2015)

| **Order** | **Family** | **Tribe** | **Taxon** |
| --- | --- | --- | --- |
| Primate | Hominidae |  | *Australopithecus sediba* |
|  | Cercopithecidae |  | *Papio hamadryas angusticeps* |
|  |  |  | *Parapapio* sp. |
| Carnivora | Canidae |  | *Vulpes skinneri* |
|  |  |  | *Vulpes* sp. |
|  |  |  | *Lycaon* sp. |
|  | Felidae |  | *Dinofelis barlowi* |
|  |  |  | *Dinofelis* sp. |
|  |  |  | *Felis silvestris* |
|  |  |  | *Felis nigripes* |
|  |  |  | *Megantereon whitei* |
|  |  |  | *Panthera pardus* |
|  |  |  | *Panthera* sp. |
|  | Hyaenidae |  | *Parahyaenna brunnea* |
| Artiodactyla | Bovidae | Alcelaphini | *Megalotragus* sp. |
|  |  | Antilopini | *Antidorcas recki* |
|  |  | Tragelaphini | *Tragelaphus strepsiceros* |
|  |  |  | *Tragelaphus scriptus* |
|  |  | Cephalophini | *Oreotragus* sp. |
|  |  | Ovibovini | *Makapania broomi* |
|  | Suidae |  | *Metridiochoerus andrewsi* |
| Perissodactyla | Equidae |  | *Equus* sp. |

**References**

Brophy JK, de Ruiter D, Fortelius M, Bamford M, and Berger LR. 2016. Pleistocene Bovidae (Mammalia) from Malapa, Gauteng province, South Africa. *Palaeontologia Electronica* 19:1 - 22.

Dirks P, Kibii JM, Kuhn BF, Steininger C, Churchill SE, Kramers JD, Pickering R, Farber DL, Meriaux A, Herries AI, King G, and Berger LR. 2010. Geological detting and age of *Australopithecus sediba* from Southern Africa. *Science* 328:205 - 208.

Hanon R, Patou-Mathis M, Pean S, and Prat S. 2019. Paleobiodiversity and large mammal associations during the Late Pliocene and the Early Pleistocene in South Africa *Quaternaire* 30:243 - 256.

Val A, Dirks P, Backwell L, d'Errico F, and Berger LR. 2015. Taphonomic analysis of the faunal assemblage associated with the hominins (*Australopithecus sediba*) from the early Pleistocene cave deposits of Malapa, South Africa. *PLoS One* 10:e0126904. 10.1371/journal.pone.0126904
