# Supplementary material for: The large mammal fossil fauna of the Cradle of Humankind, South Africa: a review
Source: PeerJ. 2025 Feb 24;13:e18946. doi: 10.7717/peerj.18946 (PMC11867040; doi:10.7717/peerj.18946)
Supplement: Supplemental Information 8 [file peerj-13-18946-s008.docx]

**Supplementary Table S8.** Taxonomic list of large mammal species at Sterkfontein. Integrated data from Hanon et al. (2019); Kibii (2004); McKee (1991); O'Regan & Reynolds (2009); Pickering et al. (2004); Reynolds & Kibii (2011); Turner (1997); Vrba (1974a); Vrba (1974b)

| **Order** | **Family** | **Tribe** | **Taxon** | **JC** | **SB** | **Mb4** | **Mb5 E** | **Mb5 W** | **StW53** |
| --- | --- | --- | --- | --- | --- | --- | --- | --- | --- |
| Primate | Hominidae |  | *Australopithecus africanus* |  |  | X |  |  | X |
|  |  |  | *Australopithecus* sp. | X | X | X |  |  |  |
|  |  |  | *Paranthropus robustus* |  |  |  | X |  |  |
|  |  |  | *Homo* sp. |  |  |  |  | X |  |
|  | Cercopithecidae | | *Cercopithecoides williamsi* |  | X | X |  |  |  |
|  |  |  | *Papio hamadryas robinsoni* |  |  | X |  |  |  |
|  |  |  | *Papio izodi* | X | X | X |  |  |  |
|  |  |  | *Papio ingens* |  |  | X |  |  |  |
|  |  |  | *Parapapio jonesi* | X | X | X |  |  |  |
|  |  |  | *Parapapio broomi* | X | X | X |  |  |  |
|  |  |  | *Parapapio whitei* |  |  | X |  |  |  |
|  |  |  | *Theropithecus oswaldi* |  |  |  | X |  | X |
| Carnivora | Canidae |  | *Canis mesomelas* | X |  | X |  | X | X |
|  |  |  | *Canis brevirostris* |  |  | X |  |  |  |
|  |  |  | *Canis antiquus* |  |  | X |  |  |  |
|  |  |  | *Canis* sp. |  |  |  | X |  |  |
|  |  |  | *Vulpes chama* | X |  |  |  |  |  |
|  |  |  | *Nyctereutes terblanchei* |  |  |  |  | X |  |
|  | Felidae |  | *Homotherium latidens* | X |  | X |  |  |  |
|  |  |  | *Homotherium crenatidens* |  |  | X |  |  |  |
|  |  |  | *Panthera pardus* | X | X | X |  |  |  |
|  |  |  | *Panthera leo* | X | X | X | X | X |  |
|  |  |  | *Panthera* sp. |  |  |  |  |  | X |
|  |  |  | *Acynonix jubatus* |  | X |  |  |  |  |
|  |  |  | *Caracal caracal* | X | X |  |  |  |  |
|  |  |  | *Dinofelis barlowi* |  | X | X |  | X |  |
|  |  |  | *Megantereon cultridens* |  | X | X |  |  |  |
|  | Hyaenidae |  | *Chasmaporthetes nitidula* | X | X | X |  |  |  |
|  |  |  | *Chasmaporthetes silberbergi* | X | X | X |  |  |  |
|  |  |  | *Chasmaporthetes* sp. | X |  | X |  |  | X |
|  |  |  | *Crocuta crocuta* |  |  | X |  | X |  |
|  |  |  | *Pachycrocuta brevirostris* |  |  | X | X |  |  |
|  |  |  | *Parahyaenna brunnea* |  |  | X |  | X |  |
|  |  |  | *Proteles* sp. |  |  |  |  | X |  |
| Artiodactyla | Bovidae | Alcelaphini | *Connochaetes taurinus* | X |  |  |  |  |  |
|  |  |  | *Connochaetes* sp. |  |  | X |  |  |  |
|  |  |  | *Megalotragus* sp. |  |  | X |  |  |  |
|  |  |  | *Damaliscus parmularius* |  |  | X |  |  |  |
|  |  |  | *Damaliscus* sp. |  |  |  | X | X | X |
|  |  |  | *Parmularius* sp. |  |  | X |  |  |  |
|  |  |  | Alcelaphini indet. |  |  |  |  | X | X |
|  |  | Antilopini | *Antidorcas recki* |  |  | X |  |  |  |
|  |  |  | *Antidorcas bondi* |  |  | X |  |  |  |
|  |  |  | *Antidorcas* sp. |  |  |  | X | X |  |
|  |  |  | *Gazella gracilior* |  |  |  |  |  | X |
|  |  |  | *Gazella* sp. |  |  | X |  |  |  |
|  |  | Aepycerotini | *Aepyceros melampus* |  |  | X |  |  |  |
|  |  |  | *Aepyceros* sp. |  |  |  |  | X |  |
|  |  | Neotragini | *Raphicerus* sp. |  |  |  | X | X |  |
|  |  | Ovicaprini | *Makapania broomi* | X | X | X |  |  | X |
|  |  |  | *Makapania* sp. |  |  | X |  |  |  |
|  |  | Bovini | *Syncerus* sp. | X |  | X |  |  |  |
|  |  | Hippotragini | *Hippotragus equinus* |  |  | X |  |  |  |
|  |  |  | *Hippotragus cookei* |  |  | X |  |  |  |
|  |  |  | *Hippotragus* sp. | X |  |  |  |  |  |
|  |  | Reduncini | *Redunca arundinum* |  |  | X |  |  |  |
|  |  |  | *Redunca darti* |  |  | X |  | X |  |
|  |  |  | *Redunca* sp. | X |  |  |  |  |  |
|  |  | Tragelaphini | *Tragelaphus strepsiceros* |  |  | X |  |  |  |
|  |  |  | *Tragelaphus angasi* |  |  | X |  |  |  |
|  |  |  | *Tragelaphus* sp. |  |  |  | X |  |  |
|  |  |  | Tragelaphini indet. | X |  |  |  | X |  |
|  |  | Peleini | *Pelea capreolus* |  |  | X |  |  |  |
|  |  | Cephalophini | *Oreotragus major* |  |  | X |  |  |  |
|  |  |  | *Oreotragus* sp. |  |  |  | X |  |  |
|  | Suidae |  | *Potamochoerus porcus* | X |  |  |  |  |  |
|  |  |  | *Metridiochoerus modestus* |  |  |  | X |  |  |
|  |  |  | *Metridiochoerus* sp. |  |  | X |  |  |  |
|  |  |  | *Phacochoerus aethiopicus* |  |  |  |  | X |  |
|  |  |  | *Phacochoerus africanus* |  |  |  |  | X |  |
| Perissodactyla | Equidae |  | *Equus capensis* |  |  | X |  |  |  |
|  |  |  | *Equus* sp. | X |  |  | X | X | X |
|  |  |  | *Hipparion lybicum* |  |  | X |  |  |  |
| Proboscidea | Elephantidae |  | *Elephas recki* |  |  | X |  |  |  |

* JC = Jakovec Cavern, SB = Silberberg Grotto, Mb5 E = Member 5 East, Mb5 W = Member 5 West, StW53 = StW53 Infill

**References**

Hanon R, Patou-Mathis M, Pean S, and Prat S. 2019. Paleobiodiversity and large mammal associations during the Late Pliocene and the Early Pleistocene in South Africa *Quaternaire* 30:243 - 256.

Kibii JM. 2004. Comparative taxonomic, taphonomic and palaeoenvironmental analysis of 4-2.3 million year old Australopithecine cave infills at Sterkfontein.

McKee JK. 1991. Palaeo-ecology of the Sterkfontein Hominids: A review and synthesis. *Palaeontologia Africana* 28:41 - 51.

O'Regan HJ, and Reynolds SC. 2009. An ecological reassessment of the southern African carnivore guild: a case study from Member 4, Sterkfontein, South Africa. *J Hum Evol* 57:212-222. 10.1016/j.jhevol.2009.04.002

Pickering TR, Clarke RJ, and Heaton JL. 2004. The context of Stw 573, an early hominid skull and skeleton from Sterkfontein Member 2: taphonomy and paleoenvironment. *Journal of Human Evolution* 46:279-297. 10.1016/j.jhevol.2003.12.001

Reynolds SC, and Kibii JM. 2011. Sterkfontein at 75: review of palaeoenvironments, fauna and archaeology from the hominin site of Sterkfontein (Gauteng Province, South Africa). *Palaeontologia Africana* 46:59 - 88.

Turner A. 1997. Further remains of Carnivora (Mammalia) from the Sterkfontein hominid site. *Palaeontologia Africana* 34:115 - 126.

Vrba E. 1974a. Description and taxonomy of the Sterkfontein extension West pit (SE) Bovidae. *Transvaal Museum Memoirs* 21:49 - 52.

Vrba E. 1974b. Description and taxonomy of the Sterkfontein type locality (STS) Bovidae. *Transvaal Museum Memoirs* 21:43 - 48.
