# Supplementary material for: The large mammal fossil fauna of the Cradle of Humankind, South Africa: a review
Source: PeerJ. 2025 Feb 24;13:e18946. doi: 10.7717/peerj.18946 (PMC11867040; doi:10.7717/peerj.18946)
Supplement: Supplemental Information 9 [file peerj-13-18946-s009.docx]

**Supplemental Table S9.** Taxonomic list of large mammal species at Swartkrans. Integrated data from de Ruiter (2003); Hanon et al. (2019); Vrba (1974a); Vrba (1974b); Watson (1993)

| **Order** | **Family** | **Tribe** | **Taxon** | **Mb1** | **Mb2** | **Mb3** |
| --- | --- | --- | --- | --- | --- | --- |
| Primate | Hominidae |  | *Paranthropus robustus* | X | X | X |
|  |  |  | *Homo* sp. | X | X |  |
|  |  |  | *Homo erectus* | X | X |  |
|  | Cercopithecidae |  | *Cercopithecoides williamsi* | X | X |  |
|  |  |  | *Cercopithecoides coronatus* | X |  |  |
|  |  |  | *Papio hamadryas* | X | X | X |
|  |  |  | *Papio ingens* | X | X |  |
|  |  |  | *Papio robinsoni* | X |  |  |
|  |  |  | *Parapapio jonesi* | X |  |  |
|  |  |  | *Papionini* sp. |  | X | X |
|  |  |  | *Theropithecus oswaldi* | X | X | X |
|  |  |  | *Gorgopithecus major* | X |  |  |
| Carnivora | Canidae |  | *Vulpes* sp. | X |  | X |
|  |  |  | *Canis mesomelas* | X | X | X |
|  | Felidae |  | *Panthera pardus* | X | X | X |
|  |  |  | *Panthera leo* | X | X |  |
|  |  |  | *Homotherium* sp. |  | X |  |
|  |  |  | *Caracal caracal* | X |  |  |
|  |  |  | *Megantereon cultridens* | X |  | X |
|  |  |  | *Felis serval* | X |  |  |
|  |  |  | *Felis lybica* | X |  | X |
|  |  |  | *Acynonix jubatus* | X | X | X |
|  |  |  | *Dinofelis* sp. | X |  |  |
|  | Hyaenidae |  | *Parahyaenna brunnea* | X | X | X |
|  |  |  | *Chasmaporthetes nitidula* | X | X | X |
|  |  |  | *Crocuta crocuta* | X | X | X |
|  |  |  | *Proteles* sp. | X | X | X |
| Artiodactyla | Bovidae | Alcelaphini | *Megalotragus* sp. | X | X | X |
|  |  |  | *Connochaetes* sp. | X | X | X |
|  |  |  | *Damaliscus* sp. | X | X | X |
|  |  |  | *Parmularius* sp. | X | X | X |
|  |  | Antilopini | *Antidorcas marsupialis* | X | X | X |
|  |  |  | *Antidorcas bondi* | X | X |  |
|  |  |  | *Antidorcas recki* | X | X | X |
|  |  |  | *Gazella* sp. | X | X | X |
|  |  | Tragelaphini | *Taurotragus oryx* |  | X | X |
|  |  |  | *Tragelaphus strepsiceros* | X | X | X |
|  |  |  | *Tragelaphus scriptus* |  | X |  |
|  |  |  | *Tragelaphus angasi* |  | X |  |
|  |  | Neotragini | *Raphicerus campestris* | X |  | X |
|  |  |  | *Ourebia ourebi* |  | X |  |
|  |  | Bovini | *Syncerus* sp. | X |  | X |
|  |  |  | *Pelorovis* sp. |  | X |  |
|  |  | Peleini | *Pelea capreolus* | X | X | X |
|  |  | Reduncini | *Redunca arundinum* | X |  |  |
|  |  |  | *Kobus leche* |  | X | X |
|  |  | Cephalophini | *Oreotragus oreotragus* | X | X | X |
|  |  | Hippotragini | *Hippotragus gigas* | X |  | X |
|  |  |  | *Hippotragus niger* |  | X | X |
|  |  |  | *Hippotragus* sp. | X |  |  |
|  |  | Ovicaprini | *Makapania* sp. | X |  |  |
|  | Giraffidae |  | *Sivatherium maurusium* |  | X |  |
|  | Suidae |  | *Metridiochoerus andrewsi* | X | X | X |
|  |  |  | *Metridiochoerus modestus* |  | X |  |
|  |  |  | *Phacochoerus* sp. | X | X | X |
|  | Hippopotamidae |  | *Hippopotamus* sp. | X | X | X |
|  | Giraffidae |  | *Sivatherium maurusium* | X |  |  |
| Perissodactyla | Equidae |  | *Equus capensis* | X | X | X |
|  |  |  | *Equus quagga* |  |  | X |
|  |  |  | *Hipparion lybicum* | X | X | X |
| Proboscidea | Elephantidae |  | *Elephas* sp. | X |  | X |

* The Lower Bank and Hanging Remnant of Member 1 are combined here due to some publications combining

their faunal lists

**References**

de Ruiter D. 2003. Revised faunal lists for Members 1-3 of Swartkrans, South Africa. *Annals of the Transvaal Museum* 40:29 - 41.

Hanon R, Patou-Mathis M, Pean S, and Prat S. 2019. Paleobiodiversity and large mammal associations during the Late Pliocene and the Early Pleistocene in South Africa *Quaternaire* 30:243 - 256.

Vrba E. 1974a. Description and taxonomy of the Swartkrans Member 1 (SKa) Bovidae. *Transvaal Museum Memoirs* 21.

Vrba E. 1974b. Description and taxonomy of the Swartkrans Member 2 (SKa) Bovidae. *Transvaal Museum Memoirs* 21:22 - 30.

Watson V. 1993. Composition of the Swartkrans bone accumulations, in terms of skeletal parts and animals represented. In: Brain CK, ed. *Swartkrans: A cave’s chronicle of early man*. Pretoria: Transvaal Museum Monograph, 35 - 74.
